# Children’s nutritional health and wellbeing in food insecure households in Europe: A qualitative meta-ethnography

**DOI:** 10.1371/journal.pone.0292178

**Published:** 2023-09-29

**Authors:** Zoë Bell, Steph Scott, Shelina Visram, Judith Rankin, Clare Bambra, Nicola Heslehurst

**Affiliations:** Population Health Sciences Institute, Newcastle University, Newcastle upon Tyne, United Kingdom; Patuakhali Science and Technology University, BANGLADESH

## Abstract

Since the 2008 global financial crisis, there has been a rise in the number of people experiencing food insecurity. Particularly vulnerable are households with children. This systematic review and meta-ethnography of qualitative studies focuses on families’ perceptions of food insecurity and how it affects children’s nutritional health and wellbeing. Six electronic databases (Medline, Scopus, Web of Science, EMBASE, CINAHL and ASSIA), were searched for studies from European high-income countries between January 2008—July 2021, and supplemented by searches of grey literature databases, relevant websites, examination of reference lists and citation searches. We adhered to PRISMA and eMERGe guidelines to improve the completeness and clarity of meta-ethnographic reporting. Methodological quality of the studies were assessed using the Critical Appraisal Skills Programme qualitative checklist. We identified 11,596 records; we included 19 publications involving 813 participants in total. Data were synthesised according to Noblit & Hare’s seven phases of meta-ethnography. We identified four key themes—food and eating practices, awareness, fragility, and networks of care–comprising five sub-themes. Our meta-ethnography provides a progressive ‘storyline’ of the children’s experiences of food insecurity from both caregivers and children’s perspectives. We found that children are aware of their family’s limited resources and are often active in trying to help their families cope, and that food insecurity adversely impacts children’s physical, psychological, and social experiences. Our analysis highlights gaps in knowledge about how food insecurity impacts children’s nutritional health and wellbeing. It suggests that future research should prioritise minoritised ethnic communities, children living in temporary accommodation and caregivers of very young children.

## Introduction

Food insecurity is defined as when “people do not have adequate physical and economic access to sufficient, safe and nutritious foods that meet their dietary needs and preferences for an active and healthy life” [1]. Further, accessing food in “socially acceptable” ways is hindered [2]. In high-income countries (HICs), food insecurity is less about quality food being broadly available but is more likely to result from poverty, unemployment and low-income; thus, low-income households are most likely to experience food insecurity due to their economic vulnerability [3–6]. In 2008, due to a global financial crisis, many European countries faced a recession, and all HICs saw increased poverty rates post the global financial crisis. More recently, the COVID-19 pandemic and ensuing rapid rise in the cost of living has had profound effects on economically vulnerable families [7], exacerbating food insecurity [8]. To varying degrees, many European HICs responded to the 2008 crisis with austerity measures [9]. Austerity did not affect everyone equally. Evidence shows that effects of austerity reforms fell disproportionately on low-income households of working age [10], with ensuing negative health impacts. An analysis of trends in infant mortality rate examined that the increasing infant mortality rate in England between 2014–2017 was disproportionately affecting the most deprived areas in the country, with more affluent areas unaffected [11]. Further, government measurements imposed during the pandemic such as lockdowns and social distancing measures affected access to food for families [8], whilst school closures increased the risk of food insecurity for vulnerable children, with schools unable to provide food like pre-pandemic times.

Not all countries systematically measure food insecurity and the evidence-base lacks a single preferred tool for its measurement resulting in different estimates of prevalence [12]. Further, with incomplete datasets it can be difficult to explore health outcomes associated with food insecurity. Existing quantitative analyses demonstrate that individuals experiencing food insecurity in HIC are more likely to have a poorer quality diet than those who are food secure [13]. For children and young people, food insecurity is less consistently associated with lower dietary quality, although there is substantial evidence of lower intake of fruit and vegetables [14]. Theories explaining this include maternal sacrifice and supplementation of food by adolescents’ who are of an age to protect themselves against food insecurity through food sources outside the home [15]. Thus, parental views of children’s and young people’s nutrition may only provide a partial picture. Further, there is evidence showing that parents are not always aware of their children’s experiences of food insecurity, with inconsistencies between parents’ and children’s perspectives of what the child is experiencing [16]. A US study investigating food insecurity and hunger from different family member’s perspectives found that children (9–16 years) described their direct experience of food insecurity in the household, whereas adults referred to the economic context in which food insecurity exists. For the success of future policies, it is important that the lived experiences of children be heard [17]. However, the inconsistent link between food insecurity and diet quality amongst children may also reflect study design. Often in studies the only measure to proxy a healthy diet is fruit and vegetable intake. This is beginning to shift with recent evidence from Canada showing a link between severity of household food insecurity and ultra-processed food intake [18]. Further research should collect more robust dietary data to fully assess the impact of food insecurity on children’s dietary quality.

Nutrition in the early years is particularly important, with the first 1001 days of life, from conception to age two, recognised as a critical period for setting the foundations of lifelong emotional and physical wellbeing [19]. It constitutes a period crucial for human development when a baby’s brain begins to grow, develop and build its foundations for health [20, 21]. Household food insecurity during this period influences child development through both direct (under-nutrition) and indirect (parenting and parent well-being) pathways [22, 23]. Key neurodevelopmental processes are impaired by inadequate intake of key nutrients such as iron, zinc, and protein. The emotional strain of food insecurity that caregivers experience can adversely influence their mental health, parenting style and child feeding practices. Although the directionality of this link is unclear, these indirect factors can adversely impact child diet quality, eating behaviours and social-emotional health [22, 23]. This is concerning given that nutrition in the early years tracks forward into adolescence and adulthood [24], with dietary patterns and food preferences remaining quite stable after the age of three to four years [24, 25]. Systematically reviewing the literature will help to fully explore the impact of food insecurity on caregivers’ infant feeding beliefs, styles, and practices.

To date, most (systematic) reviews exploring the effects of food insecurity on children’s nutrition have reviewed quantitative studies [14, 26, 27]. There is a gap in the literature for qualitative reviews on food insecurity focusing on HICs. This is despite evidence that food insecurity is a prevalent issue, raising major public health and social concerns [3, 8, 28]. There are several previous qualitative reviews of relevance set within the wider content of poverty and deprivation [17, 29, 30], published in 2005 and 2006, prior to the 2008 global financial crash. These reviews showed how the pervasiveness of poverty affects children’s social wellbeing. Of particular importance is Attree’s [29] review which explored how children perceive, react and cope living with disadvantage. Eight UK-based studies were synthesised using a meta-ethnographic approach. The review found the costs of poverty to move beyond a material experience and to be deeply social, impacting children’s social inclusion. Secondly, it identified various coping strategies used by children to deal with poverty; within the family, outside the family and applying their own resourcefulness. Thirdly, it showed how disadvantage created *‘limited horizons’* for children’s life expectations. However, this review (and others) did not explore how lack of food affects children’s physical, social and psychological health, and wellbeing.

Therefore, the aim of this qualitative systematic review and meta-ethnography was to explore and synthesise children’s experiences of nutritional health and wellbeing (from their own perspectives and those of their caregivers).

## Methods

We conducted a systematic review and meta-ethnographic synthesis of primary qualitative studies. A full protocol for this review is published [31] and registered with PROSPERO (ID: CRD42020214159). Due to large numbers of included studies, this review was carried out as part of a wider review. A sister-review exists, where the methods are fully described, focusing solely on women’s experiences [32]. Methods have been adapted here to reflect the focus on children’s nutritional health and wellbeing. The review is reported in line with PRISMA guidelines (see S1 Checklist) [33]. As this is a meta-ethnography, we also adhered to eMERGe Reporting Guidance to improve the completeness and clarity of meta-ethnographic reporting (see S1 File) [34].

### Search strategy and screening

Electronic databases (Scopus, MEDLINE, EMBASE, CINAHL, Applied Social Science Index (ASSIA) and Web of Science) were searched in March 2021 and updated in July 2021, supplemented by grey literature searches using Trove, Open Access Theses and Dissertations (OATD), OpenGrey Europe and relevant stakeholder websites. Due to limitations with database indexing when searching for qualitative research [35], supplementary searches included screening reference lists and citations for all included studies. Search terms consisted of four main concepts in accordance with the PICOS tool [36] (S2 File). Studies were imported into EndNote version X9.3.3 [37] for de-duplication, then imported into Rayyan [38] for screening. Titles, abstracts and full texts were screened by ZB and a second independent reviewer (split between SS, SV and NH). Discrepancies were resolved with a third reviewer.

### Study eligibility criteria

Primary qualitative studies reporting accounts of children’s nutritional health and wellbeing were eligible for inclusion. Nutrition-related outcomes for children included: diet (e.g., quality and quantity of food, eating behaviours, eating patterns) and food practices (e.g., involvement in food acquisition, food preparation). Nutritional health and wellbeing outcomes for children spanned physical (e.g., perspectives on their weight) and mental (e.g., anxiety about family’s finances to buy food, anxiety about food at school environment) outcomes. Additionally, eligible studies needed to report data collected between 2008 and 2021 in European OECD HICs (as defined by World Bank, see protocol [31]) and be available in the English language. Studies restricted to a specific type of population not directly related to children, or wider populations with clinical needs, that necessitate a specific diet (e.g. studies in the context of children living with HIV, type 1 diabetes etc.) were excluded. Studies were excluded if they explored the broader health impacts of poverty rather than explicitly linking health impacts directly to nutrition.

### Data extraction and quality appraisal

Data extraction used a standardised form and was extracted by one reviewer (ZB) with a sample checked by a second reviewer (split between SS, SV, NH). We used the Critical Appraisal Skills Programme (CASP) qualitative checklist [39] to assess the quality of reporting of studies. One reviewer (ZB) appraised all studies, with a sample of included studies independently double reviewed to check agreement (SS). Discussions amongst the two reviewers resolved any discrepancies. This process provided an overview of the quality of included studies for context and informed discussion of the strengths and limitations of existing evidence. Quality appraisal was not used as part of exclusion criteria but instead provided an overview of the quality of included studies for context and informed discussion of the strengths and limitations of existing evidence.

### Translating and synthesising

Meta-ethnography is a systematic approach commonly used to enable new insights into qualitative evidence from multiple studies. It is an interpretive approach that moves beyond describing or aggregating findings, instead aiming to *‘synthesise understanding’* [40]. The integration of findings from multiple studies conducted in different settings enabled our development of a deeper insight into understanding food insecurity in the context of nutritional health and wellbeing, something individual studies alone could not have provided. Meta-ethnographic synthesis was conducted in seven steps using NVivo 10 software [41]. Step one, four authors (ZB, SS, SV, NH) independently read included studies in-depth. Step two, one author (ZB) created study sub-sets, line-by-line coding and extracting of first and second order themes. A sample of papers were duplicate-coded and discussed with the review team to view the data through different perspectives (i.e., a form of investigator triangulation). Step three used a tabular form of first order themes (interpretations) and second order themes (interpretation of interpretations) with grouped studies to create *‘meta-themes’* to determine how studies were related. The fourth and fifth steps involved translating studies by checking first and second order concepts and themes against each other. Step six synthesised the translations to create a third order construct and step seven is the expression of the synthesis written up in this publication.

## Findings

**Fig 1:**
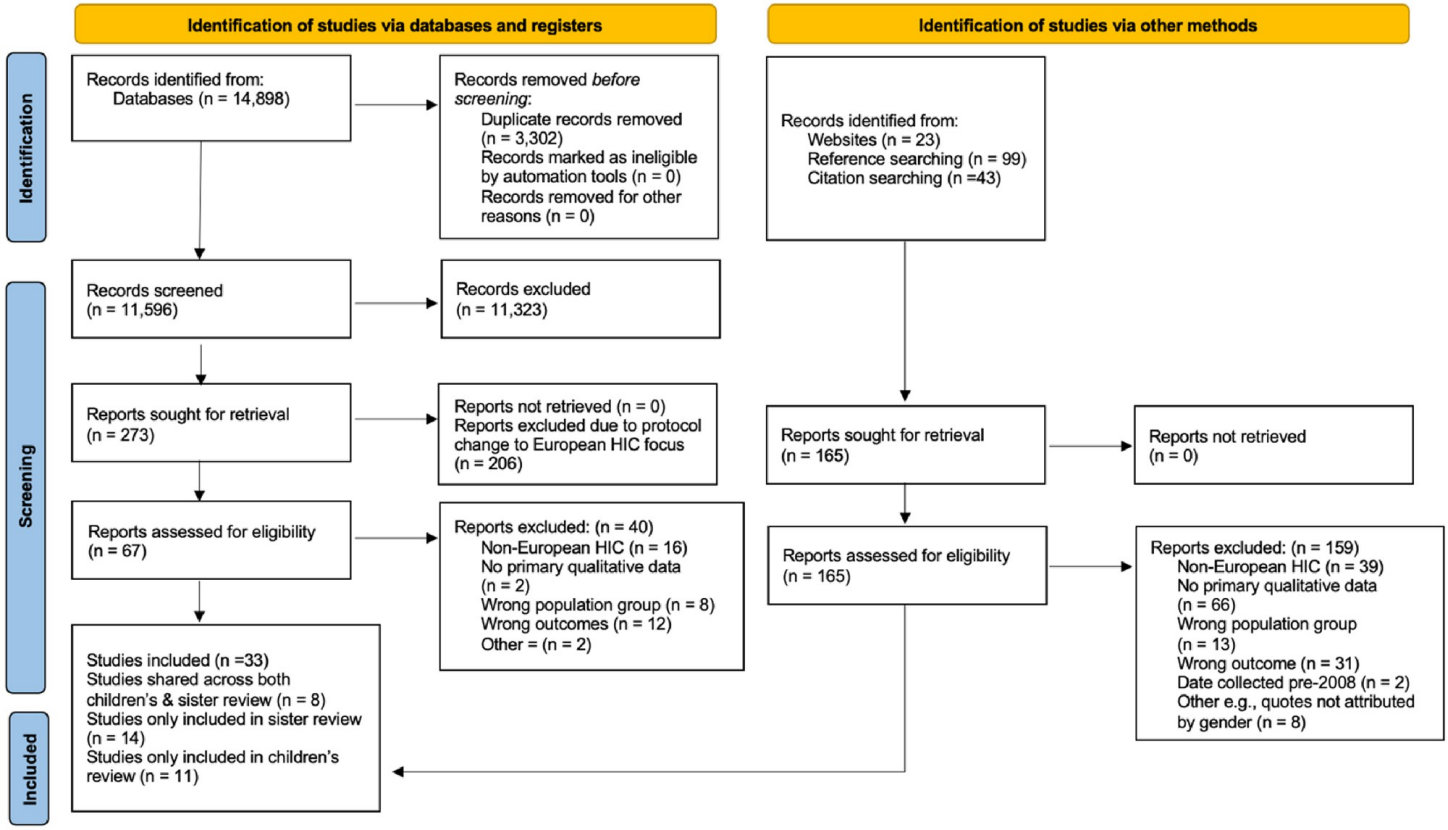
PRISMA flow diagram [33].

We identified 11,596 potentially eligible unique records through database searches, grey literature searches and stakeholder website searches, and an additional 165 from citation and reference searches. A total of 19 publications from 19 unique studies were included in the final review. Reasons for exclusion at the full-text stage are recorded and reported using a PRISMA flow diagram, Fig 1.

### Characteristics of studies

S3 File illustrates the characteristics of the 19 included studies, which represent 813 participants in total (n = 447 caregivers, n = 365 children) with sample sizes ranging from seven to 278 participants. Amongst children, ages ranged from 18 months to 17 years old. Only four studies explicitly stated the ethnicity of participants, which included white British, Norwegian, Angolian, Portuguese, Romanian, Polish, Roma, Somalian, Pakistani, Portuguese, and West African. Thirteen studies took place in England, one in Scotland, one in Ireland, one in Greece, one in Denmark and one in Spain. One study included data from multiple countries: England, Portugal, and Norway. In total, 10 studies primarily focused on food insecurity. One of these studies measured food insecurity using the 18-item United States Department of Agriculture, Household Food Security Survey Module [42], whilst five studies used either free school meal eligibility or food bank access as a proxy measure. In total, nine studies discussed food insecurity as a secondary focus in the context of wider research.

Six studies reported both caregiver and children’s perspectives, with children’s ages ranging between 10 to 15 years (S3 File). Four of these studies discussed food insecurity as the primary focus of the research, whilst the other two studies discussed food insecurity as a secondary focus in the context of wider research, for example with the primary focus being austerity or experience of a school feeding programme. Ten studies reported only the caregiver’s perspective of their children’s nutritional health and wellbeing (S3 File). Five primarily focused on food insecurity, whilst food insecurity was a secondary focus for the other five. Two of these studies focused specifically on caregivers of pre-school children, although neither had a primary focus on the impact of food insecurity on the young child’s nutritional health and wellbeing. The other eight studies reported on caregivers’ perspectives of children of all ages. Finally, three studies reported only children’s perspectives (S3 File). Children’s age ranged between 5 to 15 years, with most children between 11–15 years old. Two of these had a primary focus on food insecurity, the other a secondary focus. None of the included studies with a primary focus on food insecurity included solely caregivers of infants 0–2 years (i.e., the first 1001 days).

### Quality appraisal

Sixteen studies rated *‘high’* and *‘good’* quality, and three rated *‘low’* quality (S4 File). Studies were strong in stating clear relevant research aims, using appropriate methodologies and research design. *‘Good’* and *‘low’* scoring studies consistently scored lower by not adequately discussing reflexivity or showing how, beyond a positive ethical approval, ethics had been considered. In addition, the two lower scoring studies were reports, where reporting style differs from that of a journal article, offering less reflexivity and discussion of data analysis methods. Critically, a potential limitation of the quality appraisal process is that it assesses how studies were reported and not necessarily how they were conducted.

### Synthesis

Noblit and Hare [40] propose the notion of a *‘line of argument’*, whereby from the synthesis a storyline unfolds through the development of key themes and sub-themes. The storyline is presented in the proceeding sections through four core themes: food and eating practices, awareness, fragility, and networks of care, and five sub-themes: diet, compromised infant feeding practices, psychological fragility, social fragility, and physical fragility. Table 1 details which key themes and sub-themes developed from the data reported across included studies. Throughout the next section, direct quotations from children and their caregivers are presented in italics within quotation marks, whilst author’s interpretations are included in italics within inverted commas. Translated phrases from quotes are included in square brackets adjacent.

**Table 1:** Key themes and sub-themes emerging across included studies.

Key themes	Sub-themes	Context
**Food and eating practices**	**Diet** [43–55]	8 England, 1 Scotland, 1 Denmark, 1 Greece, 1 Ireland and 1 multi-country based* studies 8 studies caregivers’ perspective, 5 studies both children and caregivers’ perspectives, 3 studies children’s perspective 2 studies focusing on young children
	**Compromised infant feeding practices** [48, 56–59]	5 England based studies 4 caregivers’ perspective, 1 both children and caregivers’ perspectives
**Children’s awareness of food insecurity**	[46, 50, 52–55, 60]	6 England and 1 Spain based studies 2 caregivers’ perspective, 1 both children and caregivers’ perspectives, 3 children’s perspective
**Fragility**	**Psychological fragility** [46, 49, 52, 54–56, 60, 61]	6 England, 1 Greece and 1 Spain based studies 2 caregivers’ perspective, 3 both children and caregivers’ perspectives, 2 children’s’ perspective
	**Social fragility** [43, 48–50, 52–54, 56, 57]	7 England, 1 Greece and 1 multi-country based studies 2 caregivers’ perspective, 5 both children and caregivers’ perspectives, 2 children’s perspective
	**Physical fragility** [51, 52, 54, 55, 60]	3 England, 1 Spain and 1 multi-country based studies 1 caregivers’ perspective, 2 both children and caregivers’ perspectives, 2 children’s’ perspective
**Networks of care**	[43, 47, 50, 52, 55, 58, 61]	5 England and 1 multi-country based studies 3 caregivers’ perspective, 2 both children and caregivers’ perspectives, 1 children’s perspective 1 study focusing on young children

* Multi-based country includes England, Norway, and Portugal

### Theme 1: Food and eating practices

The most frequent theme to develop from the included studies related to how children’s food and eating practices were shaped by limited household food budgets. Sub-themes related to the lack of autonomy caregivers have over food choices concerning their children’s diets, how tight family food budgets shift prioritisation whilst making diet-related decisions, how these decisions become embedded in children’s day-to-day food experiences, and how food insecurity does not discriminate by age, with infant feeding practices also compromised.

#### Diet

Translating studies across one another suggests that food insecurity impacts children’s dietary variety, pattern, and overall quality, which influences their nutritional health and wellbeing (S3 File). It does so by affecting caregiver’s ability to provide regular, balanced meals that meet both the desires and nutritional needs of children. It was evident that caregivers lacked autonomy over their children’s diets, or as O’Connell and Brannen [52] say, they were unable to ‘*exercise choice’* over what food to buy and eat. Caregivers reported difficulty in consistently providing their children with meals *“It’s bad*! *For me to even have food for my kids is going to be very difficult*. *You wonder how you are going to survive tomorrow”* ([46] p.29). Breakfast was often missing, or it was not nutritionally balanced. For example, one mother talks about her children eating left-over biscuits for breakfast saying “*It doesn’t sound very good*… *but at least they’ve had something*. *I know children who go all the way to lunch without eating something”* ([43] p.38). Whilst a lone mother tells how her children rarely have breakfast and often cry for food [52]. Breakfast was more difficult for those families living in temporary accommodation who either purchased it en route to school, or received it at school if they made it on time [48]. Consistently providing meals depended on food availability in the household, which for these families reflected the cyclical nature of income; the end of the month being hardest with the most erratic dietary patterns. As one mother says *“Our eating is far more inconsistent with the way that we have to buy food now*, *so we’ll maybe have a healthy week*, *but then we’ll maybe have quite a poor nutrition week”* ([51] p.14). School holidays and weekends were also times when families felt increasing pressure on family food budgets, as were growing children who (as they got older) increased pressures on the food budget “*When he was little we’d put two potatoes on his plate*. *Now we put three or four”* ([52] p.98).

Caregivers’ accounts from England and Denmark described how food and eating practices within the household often prioritised preventing hunger rather than promoting health, despite parent’s desires to feed their children more varied, fresh and organic produce [43, 44, 47, 48]. Due to externally determined limitations constraining caregivers’ ability to *‘exercise choice’*, children were unable to be fussy, or express dislikes *“My kids will eat anything…anything cos I couldn’t afford for them to be fussy”* ([52] p.94), and missed out on nutritious foods “*You used to be able to buy lots of fruit but it’s so expensive…I just get it when I can afford it…but we need to keep the house warm…”* ([50] p.1081). Parent’s expressed concerns about their ability to provide nutritious foods, explaining that *“A typical diet is just a bellyful*, *it can’t be something where you’re gonna think healthy options*, *it’s just something to fill you up really”* ([47] p.102). Here, food was viewed as having a function to, *‘fill you up’*, rather than fulfil any culinary desires. This was expressed across the studies, with another mother sharing her difficulties in cooking even simple favourite recipes such as macaroni cheese for her daughter because *“…you need lots of cheese and cheese is a wee bit dear [expensive]”* ([45] p.62). Monotony of children’s diets was overcome by caregivers’ creativity whilst making similar dishes, keeping them interesting whilst staying within food budgets and their children’s preferences. This was crucial to avoid food waste “*You don’t have to do noodles the same way over and over*, *you know how to spice up you just get little things and put it together*. *Just make sure it’s something that you think he’d enjoy…”* ([47] p.104). For undocumented migrant families, monotonous diets included relying on staple ingredients like bread for breakfast, lunch and often dinner [52]. Lacking physical and social spaces to prepare and eat food meant children living in temporary accommodation were supplementing an already nutrient poor diet of foods such as breakfast cereal, toast, noodles, instant pizza, biscuits, and crisps with more ready meals or takeaway meals [47, 48].

For the most part, children’s personal accounts of their nutritional health and wellbeing echoed caregivers’ perspectives. There were many reports of the sufficiency of food being erratic due to money, notably at the end of the month [52–54]. From a hunger perspective, children’s experiences in terms of whether and how often they experienced hunger varied. Children described how their caregivers sometimes struggled to provide enough food to satisfy their hunger, whilst expressing gratitude for what they did have “…*even if it’s not that much food for me and [my brother] it’s enough that we’ve actually had something…”* ([52] p.96). Harvey, [54] used the term *‘problematic hunger’* to describe when hunger becomes a problem, for example when physiological hunger cannot be met with food prior to bedtime or school. Indeed, children described going to school and bed hungry, which for some occurred nearly every night or day. One child reported adopting unhealthful eating behaviours to overcome not being allowed to eat available food in the household “*Sometimes I have to sneak…Um*, *well*, *I sneak crisps…”* ([54] p.239) for which she is shamed “*we ask for too much stuff ’cos we’re hungry…*, *sometimes we just ask for too much stuff”* ([54] p.239). Whilst children across included studies did not experience *‘problematic hunger’* all the time, they consistently experienced compromised diets. Children described being unable to buy snacks “*It is difficult*, *difficult for my parents ‘cos I’ve got*, *they an’t [haven’t] got the money to give me a pound in the morning for breakfast club and then me some money for–for 30p sometimes…”* ([53] p.531) and being outpriced for fruit at the tuck shop [confectionary shop] “…*if your mum’s skint [without money] and you don’t have owt at home*, *you know*, *to take to school for fruit*, *then that’s a bit mean you’ll*, *you’ll just be hungry”* ([53] p.531). Children often conflated healthy eating with fruit and vegetables with many reporting eating below the recommended 5-a-day. Taking a wider nutritional lens, children added depth to parents’ descriptions of their diet. For undocumented migrant children they *“keep repeating the same food like over and over and over*, *just gets boring…We mostly eat rice; that’s what we mostly eat”* with others reportedly filling up on cereal and tinned rice pudding ([52] p.134). For children in temporary accommodation, eating leftovers from dinner, or bread or noodles for breakfast was the norm [54].

Across both caregiver’s and children’s accounts they described how food insecurity meant children were not able to receive *‘treats’* at all or as often as other children [43, 47, 48, 57]. *‘Treats’* were food items or meals that children would receive for a special celebratory occasion or as a reward. Lack of *‘treats’* was mainly due to cost, but for those in temporary accommodation lack of kitchen space meant children missed out on common treats such as birthday cakes as caregivers were unable to bake. On the other hand, takeaway foods were eaten more frequently for many children. Takeaway foods were bought by caregivers when no other food was in the house, and by children on the occasions when they had financial resource [54, 55]. The consumption of this energy-dense takeaway food over time raises concern about children’s nutritional health and wellbeing.

#### Compromised infant feeding practices

Caregivers described concern and worry about what they were feeding their infants. Indeed, infants in these studies (S3 File) were not afforded food security any more than others in the household. One mother described being unable to afford infant formula, therefore, ignoring doctor’s advice and giving cow’s milk despite her concerns of the impact this could have on her daughter [56]. For families living in temporary accommodation, physical and social space were additional barriers to *‘financial fragility’* for infant feeding practices [48]. For these mothers, lack of overnight kitchen access prohibited hygienic preparation and storage of infant formula, whilst for breastfeeding mothers lack of privacy and space increased difficultly of breastfeeding. Regressing a child’s diet was evident amongst those living in temporary accommodation. One mother reverted from cow’s milk to infant formula for their two-year old as they could not keep fresh cow’s milk warm in a flask and lacked kitchen access. Another mother reverted her two-year old to readymade jars for babies 4–6 months because the food on offer in the hotel was not agreeing with her son, and she felt she had limited choice [48]. These adaptive practices raise concern for the nutritional health and wellbeing of infants, potentially promoting *‘physical fragility’* (discussed in theme three).

‘*Financial fragility’* (discussed in theme three) also limited exposure to different foods. This regression of infant’s diet could inhibit development of dietary preferences. Hayter et al., [57] described what drives low-income parent’s food choices for their infants, and that food waste was a concern when on a limited budget. Parents dealt with their concerns by either not giving the child food they had previously refused or not offering foods they feared their child might not like, thereby, limiting chances of food waste “*I’ll stop buying something if they spit it out once because we don’t want the waste”* ([57] p.379). Some parents offered ready meals because “*at least then the children are going to eat it and I haven’t wasted”* ([57] p.380). However, for parents in receipt of Healthy Start Vouchers (HSV), although they were frustrated with wastage, cost did not inhibit repeatedly offering their child foods [58]. Parents were proud that their children ate fruit and vegetables, which HSV enabled them to afford “*She’s really good actually*, *because all fruit and vegetables she loves that more than if I put a plate of sausages and chips in front of her”* ([58] p.876).

During the early years, infants are reliant on their caregivers to make food and eating decisions. Parents described that their eating behaviours were reflected by their children. Parental preferences sometimes influenced what the child was offered in a potentially unhealthful way “*I don’t know about sprouts*, *I’ve never try her with sprouts*, *I don’t like them myself”* ([58] p.876), but other times this reflection prompted families to make positive health behaviour changes with some deciding to eat fewer processed snacks at night [57]. Further, migrant women showed how cultural practices shape infant feeding practices [59]. Some women described decreased duration of breastfeeding in England because of changed cultural practices, lack of privacy in crowded houses, and choosing bottle-feeding to facilitate work *“[It’s] something cultural*, *if you have a baby it’s not good for a woman to go out and the baby as well*, *so you have to keep inside and after 40 days end they have a party…[here] you have to go out”* ([59] p.459).

### Theme 2: Children’s awareness of food insecurity

There was limited evidence discussing whether caregivers thought their children were aware of food insecurity within the household; this was described in three studies which had some inconsistent views. [46, 52, 60]. Some believed their children were protected from food insecurity within the household, whilst others gave contradictory statements. For example, some caregivers from Spain believed that their child’s lack of nutritional intake was not of great concern, “*They don’t have the diet they should*, *but they are all terrain*, *like tanks*, *they are used to it”* despite their recruitment from a food bank for the study ([60] p.958). Whilst others contradicted themselves by describing how their children have never gone without, followed by statements that they cannot always feed them, *“…And sometimes you have to tell them that there is nothing to eat”* ([60] p.958) Within the same study, parents described how their children adopted active roles in the protection of the household and were frustrated when they could not help “*She feels impotent because she cannot help*. *She has cried a lot”* ([60] p.958). Indeed, caregivers across countries spoke of how living in poverty made their children mature sooner and develop self-sufficiency because of their felt responsibilities towards the family life. However, these inconsistencies highlight the need for further research speaking directly with children about their awareness of food insecurity [52, 60].

Protected or not, children’s own accounts demonstrated they were aware of food insecurity within the household, and the wider poverty context. Children described parental sacrifices, strategising, limited food supplies in the household, and were engaged in conversations about fairness around food pricing and marginalisation of those with lower income. Notably, children tended to focus on the immediate food environment, food quantity and types of food, survival rather than thriving, as one mother says *“…They are thinking about how they are surviving the next day”* [46, 54]. Some children moderated their needs when they realised there was less food in the house “…*this month I won’t ask for much”* ([52] p.120). Whilst another child speaks of how she sacrifices her own food intake to share with her mum *“I skip meals to share with my mum [inaudible]…for example*, *I skip my meal to wait for her to come back and at least we can have the same amount of food…[We] starve together through the whole day*, *so at least we will have had something to eat”* ([52] p.224). Through their own experiences of deprivation, young children were not only active participants within the family but also with their external network, forming their own networks of care.

### Theme 3: Fragility

The third theme related to how food insecurity permeates children’s nutritional health and wellbeing irrespective of parental attempts to protect their children’s food and eating practices. This includes how financial insecurity (discussed in theme one) in children’s day-to-day lives elicits fragilities around children’s psychological, social, and physical health and wellbeing.

#### Psychological fragility

Children living in food insecure households were, by virtue, living within the wider context of poverty. This context of financial insecurity impacted on children’s diet directly, but it also impacted on children’s nutritional health and wellbeing through more hidden pathways, highlighting how fragile some aspects of their everyday lives were. Hall and Perry [56] use the term *‘fragilities’* to describe where children are vulnerable to poverties injustice. They propose that *‘financial fragility’* is the root cause of other fragilities, including emotional or psychological, which undermines quality of family life. One fragile dimension of food insecure children’s lives was their emotional and psychological health. For children living in a household with ongoing economic problems, household tension was found within the included studies to be higher. Caregivers across Europe spoke of how, when money was tight, it was a constant source of stress and anxiety for them which was evidently passed onto children who they described as experiencing sadness and anxiousness about the lack of food [46, 56, 60]. Children were aware of how *‘financial fragility’* impacted household tensions *“When dad’s work don’t get his hours right or when it’s pay day we all get tense in case there are any changes…We’re on a tight income as it is and it gets quite stressful”* ([56] p.16). This was despite parental attempts to reduce emotional suffering “*If you are sad*, *that’s what your children are going to be*: *sad*. *But at home I smile*, *I live laughing*, *and that’s what they see—Sometimes I try to create another environment at home*, *but I can’t*. *Your life*, *your home*, *everything has been destroyed”* ([60] p.958). Children also internalised some of this household tension as feelings of burden, guilt and for some emotional numbness. For example, speaking of how school feeding initiatives help, one child from Greece referred to herself as a burden on her family due to her need to be fed *“My mum now doesn’t spend money for the school snack or for milk*. *I am now less of a burden on my parents…”* ([49] p.273). Whilst another child spoke of feeling guilty for eating because her mum hadn’t, *“…it gets a bit to the point where we’ll start feeling guilty because Mum hasn’t had anything and we’ve had it”* (p.96) although her mum tries to contradict this statement, saying *“I’m a warrior*, *though*. *I’m all right”* to protect her child from emotional suffering ([52] p.96). For other children, where hunger was the norm, loss of interest in food was apparent, perhaps indicating apathy or emotional numbness.

#### Social fragility

Another fragile dimension of food insecure children’s lives are their social relationships. Across the studies, food was co-constructive of care through both nutrition and social relationships. Being unable to afford sufficient food and participate in socially accepted food and eating practices was a hidden exclusionary pathway impacting children’s nutritional health and wellbeing. Starting within the family, children growing up in temporary accommodation had socially diminished circumstances, without opportunities for commensality with their families. Children’s family settings were not conducive to *‘normalised’* dining. Children dined on the bed or on the floor, parents improvising with a cloth as a table *“There is no chair to sit on*, *you have to sit on your bed*, *eat on your bed*, *do you know what I mean like*, *your bed is the focal point of your room*, *it takes up the most space”* ([48] p.145). Social participation around food was further limited as caregivers curtailed friend’s visiting children’s houses due to parental fear of being unable to feed extra people [43, 50, 52].

Outside of the family environment exclusionary pathways continued, this time in front of peers, therefore creating a greater potential for stigma and shame. Children from England described a sense of exclusion whilst waiting outside the shop for friends saying it *“Feels like I’m left out of the fun that happens and stuff*. *Like it just makes me feel empty…It makes me feel like what have I done like*, *what have I done*?*”* ([52] p.135). At school, children discussed exclusion from tuck shop foods because of the rising costs and the implications on themselves when they brought in non-permitted food items as a result *“… everyone just tells on me because my mum di’n’t have any fruit money and so I asked to see if I could bring a couple of sherbet lemons in and I got one out*, *you know*, *to quickly have*, *and erm but everyone just kept telling on me”* ([53] p.531).

Another potential exclusionary pathway in schools occurs during lunchtime, although this starts within the household. Hall et al., [43] found that a tension is encountered when deciding between school or packed lunches, with compromises made between children’s wants and affordability. Children tended to prefer parent’s home cooking or a packed lunch [43, 52, 54] and some parents restricted their food intake to provide a packed lunch. However, not all parents were able to, and for those children who did not eat their school meals, they went hungry [43, 54]. Children preferred packed lunches because they reduced stigma and isolation by peers and reduced the embarrassment from visibly having a lack of money at school. Children in England described how school meals meant missing out on eating lunch with friends who had brought in packed lunches because they were separated by meal type. Whilst for other children the limited choice of food within the free school limit of £2.20 per day could be humiliating if picking an option not suitable for the limit and being told so in front of peers. Caregiver’s, however, tended to prefer children having school meals because of a lack of affordability of an extra meal and *“…because I don’t know if I’ll have dinner…”* ([52] p.215). Indeed, some children also described preferring school meals. These children showed awareness of the benefit of free school meals (FSM), *“It’s just that my mum an’t got enough money and with four boys”* ([53] p.531) and expressed how they were a weekly treat because of affordability *“I mean*, *I can only have ‘em once a week ‘cos my mum can’t afford it”* ([53] p.531). Further, Dalma et al., [49] finds that caregivers and children in Greece benefitted from universal provision of FSM, which avoided stigma. Caregivers were relieved of tension and compromise, no longer having to afford a second meal or provide snacks for their children, whilst children were relieved of hunger *“Now with the Program I’m not hungry*. *I have something to eat”* ([49] p.273). For Somalian migrant children living in Norway, FSM were not an option with packed lunch the norm. However, for these children visibility of poverty arose if they brought in traditional cuisine which resulted in exclusionary responses by other children, as their food might be called *‘smelly’* by others. Therefore, children preferred sandwiches which were deemed more expensive to make by parents than traditional Somalian food [52].

#### Physical fragility

Another dimension that food insecurity made fragile was children’s physical health and wellbeing. Eating a sub-optimal diet, by caregivers’ standards, had knock-on digestive impacts for children, as one mother explains *“My daughter’s been quite constipated recently*, *which she’s never been like that and that’s no good for her”* ([51] p.41). *‘Problematic hunger’* was a concern for children experiencing more severe food insecurity. From five years old, children were able to describe feeling hungry and going to bed on an empty tummy. Children aged seven onwards were able to describe the physiological sensations of hunger saying things like, *“I feel sick”* ([54] p.241), *“your belly hurts and you feel sick”* ([54] p.241), *“my tummy’s aching”* ([54] p.241), or *“belly hurts and feel like vomit”* ([54] p.241). For others it provoked an emotional reaction making them feel sad and annoyed, one child wanted the feeling to go away so goes to bed *“I feel hungry*. *I just want to sleep ’cos when you sleep…when I [go] to bed hungry and sleep*, *I’m not hungry”* ([54] p.241) and where hunger was the norm for one child who goes without food the whole day, there was a dismissal of the sensation, marking a loss of interest in food *“it doesn’t bother me*, *as I said I never feel hungry”* ([54] p.241). However, few studies related these sensations directly to physical health and wellbeing impacts. Of those that did, one teenage girl was diagnosed with anaemia after showing physiological signs of *‘problematic hunger’*. She described eating *“nothing*, *a sip of Lucozade”* ([55] p.717) for lunch and *“nothing”* ([55]p.717) for dinner whilst grabbing her stomach complaining *“I feel nauseous”* ([55] p.717) stating that this had been going on for about a year. A boy living in an undocumented migrant family also suffered *‘problematic hunger’* giving rise to extreme stomach pains *“I was so hungry and that*, *so…all of a sudden yeah it was like…it was like…it was like I got hit on my belly…when I don’t eat yeah it comes*. *Yeah*, *so I’m scared that it might come back…it was like I got stabbed with a knife and it’s still there”* ([52] p.135). It left him feeling lethargic and sleepy which impacted his ability to perform at school *“Sometimes you don’t have enough energy; you cannot cope in the classroom so you have to like try and rest a bit*. *You just put your head on the table and you end up falling asleep in the classroom and you get in trouble for it"* ([52] p.135). Only one study discussed the weight status of children. This study was set in Spain with caregivers accessing food banks [60]. They reported sub-optimal growth in many children manifesting as a weight status that would indicate overweight, obesity or stunting [60].

### Theme 4: Networks of care

**Fig 2:**
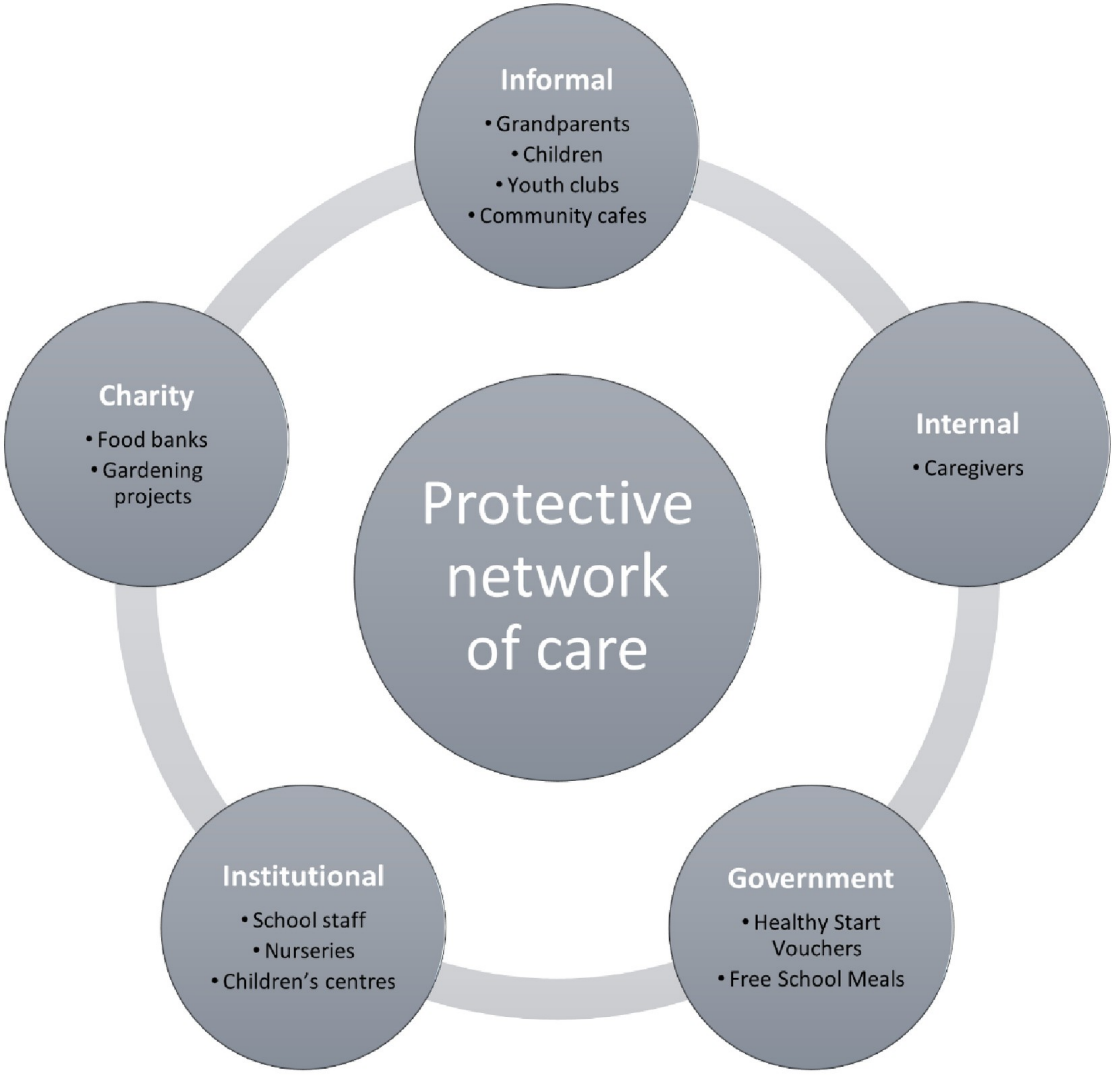
Illustration of the protective network of care safeguarding children from food insecurity.

Across the studies, various actors engaged in sacrificial or reciprocal practices to protect children from food insecurity and its impact on their eating and social practices around food. Informal support networks were identified. Grandparents helped directly by offering food and money for food, or indirectly, providing access to larger supermarkets where products are cheaper [43]. They also offered a place to introduce infants to a wider variety of foods (along with nurseries, toddler groups, and children’s centres), which parents could then incorporate into meals at home without fear of refusal or waste. Further, one aunt had her nephew sleep and eat at her house because of the lack of space and money for food at his mums, although the boy did not admit that he has experienced going without food at home [52]. Friends tended to share knowledge of cheap recipes between themselves that children might enjoy [43]. For migrant families, a mixed picture was found; many engaged in these practices, but others avoided them in fear that news of their situation would spread or because they then felt indebted to others, and did not want to be seen as begging [47, 52]. Fig 2 illustrates the protective network of care for children evident from included studies described here and in the next few paragraphs.

Children’s networks of care extended to external groups where food continued to be a vehicle through which care was shown, given, and received. Critical in this external network were schools. The role of schools went beyond delivering the service of FSM with kitchen staff helping hungry children access more food. Caregiver’s saying, *“If I ask the nuns to fortify their snacks*, *they go with them to the kitchen alone…and sometimes they give something more*. *They give them soup at the afternoon and then…I don’t worry as much”* ([52] p.216). Whilst children themselves asked for extra food, *“He was hungry*, *yes…he said that often he’d get there and the school staff*, *he’d ask the staff for bread*, *to get something”* ([52] p.140). Beyond school, youth workers were part of the network of care for young people, cooking and providing food initially from their own pockets, but later creating meals from food donated by local supermarkets [55]. Children themselves also practiced these reciprocity strategies. Laverty [55] uses the term *‘materialities of care’* to describe how food was more than nutrition but was active and co-constructive of care through both nutrition and social relationships. Boys attending a youth centre brought in takeaway food drawing visibility and opening conversations for others to engage in, *“what have you got*?*”*, *“what are you eating*?*”* ([55] p.716). Although these boys had little spending power, when they did have money, bringing in food was a way of being seen. It was also a moment to share food with others, in recognition that *“sharing is caring lad”* with boys excluded from the group if they did not participate in this reciprocity strategy ([55] p.715). Amongst the girls, however, they did not like sharing food, reporting concerns about the quality of the food shared (mainly hot food takeaways) and its impact on their weight, with one girl stating she doesn’t want a *‘fat belly’* [55]. The girls were also encouraged to provide care by cooking for the boys with female youth workers, they often refused.

Other services extending care to children through food were food banks who provided families with food parcels and the Healthy Start scheme that helped parents increase infants’ dietary quality [52, 58, 61]. Some mothers suggested a need for *“…more community cafes*, *ones that are large and welcoming enough for families”* ([61] p.9) and gardening projects as spaces for families to attend that could supplement their children’s diets and improve commensality [47].

## Discussion

This review provides a progressive storyline of the family unit’s perspective of children’s experiences of food insecurity and the ways in which it: prevents caregivers from meeting children’s nutritional needs and desires; is embedded into children’s everyday food and eating practices; (in)visibly impacts children’s psychological, social and physical health and wellbeing. Furthermore, children were found to be active in reciprocal practices of care both within and outside of the family unit. The discussion that follows considers the analysis in relation to the original aim of the review and the broader literature.

### Awareness of food insecurity within the household

Previous literature [29, 30, 62] shows that children are aware of their disadvantage in relation to others and actively engaged in managing and meditating the impacts of poverty beyond themselves. This meta-ethnography builds on this evidence showing that children are active in managing food and eating practices, both within and outside of the household. Most children actively asked for less food at home, helped parents whilst they worked extra shifts by acting as carers for siblings, split their meals with caregivers and siblings or asked for more food in places outside the home to manage and mediate the impacts of food insecurity. In this review, children as young as five years old were able to describe their *‘problematic hunger’* whilst children aged seven years were able to communicate the contextual household’s situation of a lack of food. However, although some children knew their caregivers had little money and employed practices to ensure they had enough to eat, poverty was a concept that was abstract, with references to poverty like that seen in under-developed countries. Further, a temporal dimension was evident in relation to children’s awareness of food insecurity. Children described concerns about the immediate food environment rather than future-bound worries. This resonates with previous work by Wills et al., [63] who used Bourdieu’s concept of habitus to explore how class becomes embodied in food and eating practices. Habitus is ‘*an overarching system for classifying practices behind the conditions of all lifestyles*’ which lends itself useful to analysing food and eating practices ([63] p.727). Wills et al., [63] found that working class parents were more concerned with food and eating in the present versus future-oriented concerns of middle-class parents. This played out as family ideals, with present orientation functioning as figuring out what needed to be done today. These parental practices and beliefs can be reproduced by children and transmitted down generations which has implications for considering inter-generational poverty and insecurity.

Over the past 25 years, research involving children as active participants has grown, with children recognised as *‘social agents’* [64]. Research from the USA shows that children were aware of food insecurity describing worry, sadness or anger as well as physical symptoms relating to lack of sufficient food [16]. Despite lack of familial conversation on the topic of food insecurity, some children took responsibility for managing family resources, whilst others were protected by being unaware of the issue [16]. This review finds that, within the European HIC context, children are competent to make sense of the adult world [64], reflexively acting when seeing their parents go without food or hearing them commenting about insufficiencies in the household. In this way, children do contribute and make a difference to the food and eating practices within the household. This review found that studies including children’s voices mainly focused on young persons of secondary school age (11–16 years). Potential reasons for this could be because young persons have more advanced abilities to articulate their experiences than younger children, they are at a stage of rapid personal and social growth that can be enacted through food and eating practices and they are able to take on more responsibility in the family [52, 63]. However, further studies with younger children would be useful to understand how food insecurity impacts nutritional health and wellbeing across ages.

Children’s own voices are also essential in health research as a conflicting picture developed when caregivers gave their perspective of their children’s experience of food insecurity. Some caregivers dismissed that the poor nutritional intake of their children was problematic because their children were *‘used to it’* whilst others contradicted themselves saying their children had never gone without, followed by statements telling their children they had nothing to eat. The range of responses perhaps reflects parental anxieties that consequences will arise from sharing their experience with household food insecurity, for example, that social services might get involved. As Dowler articulated in an interview, food insecurity “*…is an issue of private shame […] people keep to themselves*. *And it is an issue of private suffering”* ([65] pg. 1). In the same light, some children dismissed food insecurity as an issue stating that hunger doesn’t bother them, they never feel hungry and that they are always fed despite parental accounts detailing otherwise. This could signify that those children have recognised this is not a public issue, thus protecting their family from stigma and shame. This finding supports the need for further research including the whole family’s perspectives.

### Nutritional health and wellbeing impacts of being unable to access sufficient food

The first 1001 days is a time when infants are most at risk of the nutritional health and wellbeing impacts of food insecurity. This review shows, food insecurity does not discriminate by age, with infants’ food practices also compromised and highlights a lack of evidence specifically in first 1001 days with only England-based studies included. Those studies showed how food insecurity means babies and infants are receiving inadequate and unbalanced diets. Caregivers did not have full autonomy over their feeding practices. Being food insecure meant some babies progressed too quickly to drinking cow’s milk instead of infant formula or breastmilk, whilst older infants regressed to drinking infant formula or moved back to eating baby food. In some families where food waste was a concern, infants were either not re-offered foods they had previously refused or were not offered a variety of foods at all. Offering infants cow’s milk prior to beginning complimentary feeding can impact their growth and development as it does not contain sufficient iron content to cover baby’s needs [66]. Longitudinal studies show how either nutrient poor or nutrient rich food preferences track from infancy into childhood and adolescence [24] and that infants of two to three years are most likely to accept new foods, with dietary patterns and food preferences remaining quite stable after the age of three to four years [24, 25]. Indeed, infants start to develop flavour preferences whilst in the womb, tasting their mother’s amniotic fluid [25]. Studies have shown that a mother’s diet in the third trimester of pregnancy or whilst breastfeeding helps the infant transition to solid foods, accepting foods eaten by their mothers, with infants who are breastfed being less picky and more willing to try new foods during childhood [67, 68]. One explanation for this is that the breastmilk provides them with a sensory experience of food flavours, starting the learning process earlier on in infancy, prior to complimentary feeding starting [25]. This emphasises the importance of providing support to food insecure families to enable them to be able to repeatedly offer a variety of healthy foods during infancy as these feeding practices are important determinants of the quality of adult diets. It also highlights the need for more research amongst the first 1001 days.

Further, in this review, migrant women noted how cultural practices shaped breastfeeding practices. They described reduced breastfeeding since moving to England because of work, family, and societal pressures which reduce the amount of rest available to them and time to dedicate to breastfeeding. One approach to mitigate this would be to improve the adequacy of universal maternity cover to ensure that children’s and caregivers’ nutritional health and wellbeing is prioritised. In England, the Healthy Start scheme was found to meditate the impact of food insecurity upon infants. Healthy Start vouchers are a cash-benefit accessible for families on low-income with children 0–4 years for purchasing fruit, vegetables, and cow’s milk. In this review, the scheme provided a nutritional safety net enabling parents to repeatedly offer their children foods given that they had financial support. Other participants in this review accessed food banks. Food banks are a prominent feature within Europe, the European Food Bank Federation established in 1986 now has 330 active food banks in 29 European countries [69]. Since the 2008 global recession Spain and Portugal have seen an expansion of services alongside need, whilst in the UK this occurred in 2010 [70]. Some food banks accept donations for infant formula, providing this to families with babies in an attempt to support them. Whilst admirable, this can be a risky practice potentially unintentionally creating harm [71, 72]. Food banks are not places to make decisions about infant feeding given the lack of healthcare professionals present to help caregivers make informed decisions about infant formula. There may also be an unintended consequence reducing the prevalence of breastfeeding, the healthiest form of infant feeding [71]. Since 2014, the UN have shared their concerns providing recommendations which avoids food banks providing infant formula to families with babies [72].

This review shows that food insecurity is embedded in children’s day-to-day lives, a similar finding to reviews from pre-2008 showing how poverty and low-income impacts children’s everyday lives [29, 30, 62]. However, unlike those reviews which focused on the economic, social, and relational constraints, this review focused on food. Food insecurity affected children’s’ dietary quality and quantity. Their diets were described as meagre, monotonous, and less nutritionally dense with hot food takeaways and ready meals commonplace. Although this review had limited accounts linking diet quality and quantity with weight status, we identified how food insecurity can lead to overconsumption of energy dense and nutritionally poor foods. This happened as most caregivers and children had limited options other than to opt for foods necessary to keep full rather than focusing on nutrient-dense, balanced meals. As said by a youth worker *“what’s the point in giving soup to someone starving*?*”* ([55] p.716). For children living in temporary accommodation, the lack of access to cooking facilities and storage facilities heightened the tendency to substitute healthy food and lean on consumption of cheap, high-energy, high-processed foods [18]. With little price variation compared to nutritious foods these items make planning on a tight budget easier. Also, their potentially increased availability in areas where food insecurity is higher could partly explain the increased risk of obesity in adulthood for children experiencing food insecurity [73]. Dietary behaviours learnt through childhood will make it harder for children to engage in healthy behaviours necessary for weight loss and maintenance [74]. Indeed, a girl in this review was shamed by her parents for being hungry and responded by secretly eating food in private. Whilst young female youth centre attendees did not partake in networks of care using food or eat in public and were vigilant about the impact of hot food takeaways on their weight. These behavioural responses raise concerns about the development of eating disorders given that common signs and symptoms of eating disorders include feelings of shame and stigma around food, preoccupation with food, weight and body image, and eating in secrecy [75]. A review of emerging evidence of food insecurity and eating disorders found that, among adults, food insecurity is cross-sectionally associated with higher levels of eating disorders; however, the same robust relationship was not found amongst adolescents. This could be because fewer studies have been conducted in adolescents to date [75].

Prolonged consumption of the dietary quality and quantity seen in this review will have other nutritional health and wellbeing impacts. The inability to afford ingredients like fruit and vegetables, meat, and cheese along with the monotony of eating staple ingredients increases the risk of deficiency of micronutrients leading to illnesses such as scurvy caused by a lack of vitamin C. Poverty and health are inextricably linked; malnutrition adversely impacts physiological and mental health capacities, reducing productivity, in turn making individuals more susceptible to poverty [76]. This review builds on research from pre-2008 by showing how lack of food specifically impacted children’s everyday experiences of disadvantage [17, 29, 62]. In this review, children who went to school hungry were unable to perform at school, lacking concentration and energy. Food insecurity could therefore make it harder for children to achieve their grades, in turn making it harder to secure employment and a high-earning wage, making it harder to break the cycle of poverty [76]. Food banks attempt to reduce the number of meals skipped by children by providing emergency food parcels to families, which despite being nutritionally inadequate, help mitigate everyday adversity faced by children [77]. Food banks were set-up to provide short-term relief in emergencies, they are not a long-term solution. Food is not the root problem of food insecurity; rather it is a lack of income [78]. Thus, a suitable response requires supporting families to create sustainable livelihoods and protecting those who unable to. It requires that Governments across Europe, and wider, enshrine and enact the human right to food [2, 6, 79, 80]. Free school meal offerings varied across countries included in this review. Of those offering free or subsidised lunches, acceptance amongst children was mixed, whilst caregivers reported favorably of them, expressing how they reduced pressure on the family food budget. Children spend a considerable amount of time at school. They consume at least one meal per day there making schools an ideal setting to promote consumption of healthy food early in life. There is a potential for universal FSM to reduce the socioeconomic differences in diet and health outcomes amongst children. In the UK, a study examined the Universal Infant Free School Meals programme which offers children in the first three years of school a free lunch [81]. Positively, it found that the greatest change on diet and nutritional intakes occurred in low-income children. However, it did not observe a change in consumption of fruit and vegetables, or sugar-sweetened beverages, or dietary intake of sugar across all children, suggesting room for improvement in the quality of FSM. A non-randomised study in Norway provided FSM to children aged 10–12 years for a year and found an increase in children’s intake of healthy foods, especially amongst low-income children [82]. These studies show that advocating for universal FSM could help reduce health inequalities and stigma associated with FSM amongst children, and as this review suggests there is a need to improve children’s experiences with FSM.

This review is contextualized by a decade of austerity policies in which more deprived areas have unequally been affected [83–85] and households with children have become increasingly vulnerable to food insecurity. More recently a pandemic and now a cost of living crisis mean that household budgets are further squeezed, with food the flexible part of the budget [86]. Synthesis of included studies revealed how within this context, food insecurity impacts caregiver’s ability to *‘exercise choice’* over their children’s food and eating practices due to lack of income, physical and social space. Children’s food and eating practices thus prioritised preventing hunger rather than promoting health. Wills et al., [63] exemplify how examining routinely eaten foods can highlight the social, cultural, and economic capital families have. In this review, children’s cultural capital through food was limited as they were unable to experience cooking and tasting of more exotic forms of cuisine both at home and in restaurants. Their social capital is also minimised by lack of commensality, with food kept an internal event within the family, within the home. Indeed, food was predominantly a matter of fuel rather than enjoyment. This was evident through descriptions of limited opportunities for children to explore their dietary preferences, through parents saying how *“A typical diet is just a bellyful…”* ([47] p.102) and through parent’s limited purchasing power to try different foods, cook foods in different ways or try different flavour combinations. Instead, a monotonous diet was standard for children, based on staples or less nutritionally dense meals such as hot food takeaways and ready meals. Additionally, eating out at restaurants and trying different cuisines was limited with meals based on food in cupboards.

Studies revealed the deep vulnerabilities food insecurity exposes within children’s lives. Within the home setting, tensions were high, placing an emotional toll on all children. They experienced this as stress, anxiety, depression, sadness, and annoyance. Indeed, the same child often expressed multiple emotions within the same study, suggesting that psychological distress could worsen dependent on severity [87]. Caregivers attempted to protect their children and reduce their emotional suffering. However, children’s awareness of their relative scarcity along with household tension meant instead that some children internalised feelings of guilt, lost interest in food, and became emotionally numb. Whilst for other children, their worry meant they reduced their meal size to share with their caregivers or siblings, they asked for food at school, or engaged in networks of care. This shows how children’s adaptive coping strategies for food insecurity differ. Either they engaged with the stressful event in what Evans et al., [88] call engagement coping, or they disengaged from the stressful event, called disengaging coping. The former means that children regulated their emotions to cope with the stressor in turn increasing appreciation for their caregivers. The latter means that children withdraw or avoid the stressor and is associated with poorer mental health and depression. This finding is similar to what Attree [29] found that children either resigned to living in poverty, making do with what resources they had, or they became active agents in coping with poverty, attempting to shield their parents or family. Either approach potentially limiting children’s horizons.

The psychological impacts from food insecurity affected children’s social interactions and relationships. This supports previous reviews showing how poverty more broadly impacts children’s everyday practices [17, 29, 62]. Ridge’s review [17] including studies until 2008 similarly found that through pathways of social exclusion, or insecure social integration, poverty can have a damaging effect on school careers and children’s everyday lives at school. This review supports evidence from pre-2008, as it shows how disadvantage restricted children’s everyday food practices, rendering them aware of their difference compared with more affluent peers. The inability to partake in packed lunches, buying foods from the shop or tuck shop, eating with friends at school, inviting friends’ home or consuming desired foods were all examples of how social exclusion occurred through food and eating practices. Humans are social creatures wanting to form relationships, be part of a social groups and participate in activities with others. Building social networks increases social connections and security thus building a sense of wellbeing and belonging [89]. However, being unable to fully participate in these everyday food practices limited children’s opportunity for creating social capital. As Ridge ([17] pg. 76) says insecurity and uncertainty “*can penetrate deep into social and interpersonal relationships*, *sapping self-esteem and undermining children’s confidence*”.

From the synthesis, caregivers and children described networks of care that attempted to reduce the impact of food insecurity for children. Like Attree’s reviews [29, 30] networks included those within the family, outside the family and children’s own resourcefulness. However, arguably, since 2008 the dependency on external networks has strengthened as austerity policies stripped away social services including youth centres, Sure Start centres alongside increasing costs of childcare; the UK is amongst the highest in OECD countries for childcare costs [90]. Moreover, in this review that focused on food, food was more than nutrition, but a vehicle for giving and receiving care for the body through social relationships [91]. Using Tronto’s [92] four central practices of care we can begin to explore how informal support networks of care come together for children. As Tronto [92] says, care arises from the fact that, as humans, we are not always able to take of ourselves. Caregivers in this review were not always able to take care of their children, and children, given their age, were not able to take full care of themselves either. For this reason, other people step into a caring role. Tronto [92] suggests four elements to care. They are caring about (noticing the need to care), taking care of (taking responsibility of care), caregiving (doing the actual work needed to care), and care-receiving (the response to care). From these, four ethical elements of care arise: attentiveness, responsibility, competence, and responsiveness. Throughout the review, it was evident that different groups of people acted in different ways to show care through food. Children were responsive to the care provided but also active in the role of caring about, taking care of, and caregiving. Within their own social groups, with the little money they had, boys exercised an ethics of care approach by sharing food amongst themselves. Caregivers exercised three of the four elements of care being attentive to the need for care, taking responsibility for caring and doing the work to care for their children. Youth centres were also attentive to the need for care, recognising those children in attendance who were experiencing food insecurity, and doing the work to care for their children by providing money to buy food, or cooking meals from arranged donations of food. School staff similarly recognised children in need offering extra food to those children in addition to FSM. Informal support from grandparents, community cafes, food banks, gardening projects, children’s centres, nurseries, and toddler groups also showed ways of caregiving. Either directly providing food to families or indirectly helping them access more food. This myriad of support comes together to help families with children cope with economic adversity. Children with no or inconsistent access to these settings are further marginalised with repsect to accessing food.

### Strengths and limitations

This review is the first to synthesise qualitative data from across European HICs on the impact of food insecurity on children’s nutritional health and wellbeing. This review is the first to explore these experiences post 2008 global financial crises, a period which set poverty trajectories to increase, and food insecurity to worsen. Indeed, to our knowledge, no other published systematic review, focuses on the family unit, including both caregivers and children’s perspectives in its analysis. A strength of this review is the inclusion of children’s own perspectives alongside caregivers. With respect to children and young people, most health research has been based on parent’s, caregivers’, or stakeholders’ views. A limitation of using their perceptions rather than directly asking children lies in the recognition that older children eat outside of the home and therefore parent’s views might not have a true picture of their nutrition. Another key strength of this review is its meta-ethnographic approach to synthesis of review findings, driven by both participant experiences and third order author interpretations. This enabled development of a *‘line of synthesis’* moving beyond the individual studies *to ‘more than the sum of its parts’* [93]. A common limitation associated with meta-ethnographies is a reliance on the original study author’s pre-selected participant quotes and interpretation of the data in published articles from which the review author generates a *‘line of synthesis’* [35], and our review is also limited by this factor. However, to keep the synthesis grounded in participant experiences, we have presented original quotes throughout the findings. Rigorous gold standard methodologies were used to develop the protocol (PRISMA-P) conduct the review (PRISMA) [33] and report the findings (e-MERGE) [34]. Recognising the limitations of database searches in retrieving qualitative literature, grey literature, stakeholder websites and reference and citation screening was included as part of a comprehensive search strategy.

This review also has several limitations. Like other reviews, it is possible for this review to be subject to publication bias, whereby studies are not published if they do not show clear or marked results [94]. We attempted to overcome this by searching theses databases. In this review, one study used the USDA survey to measure of food insecurity, whilst many others used socioeconomic status or proxy measures like FSM or food bank use. This might be a product of the qualitative nature of studies, however, not using a specific measure will likely impact the experiences of food insecurity captured. For example, it may only include the experiences of those accessing services, missing the voices of those who are food insecure but who could not access or did not know about services. An additional potential limitation (but also strength) of this review is the diverse range of included studies, from different European contexts, where contextual factors could impact the experiences of the family unit. Patterns of food insecurity across countries and welfare regimes are varied by welfare regime, with the UK and Republic of Ireland seeing the sharpest rise [3]. While this variation potentially limits the ability to draw meaningful conclusions from the studies, the diversity of included studies has enabled exploration of perspectives from a broad spectrum of family’s experiences of food insecurity, which has demonstrated common experiences from within and across the themes. This review has shown that despite different social security systems and different economic, social, and cultural contexts, families are reporting similar qualitative experiences for children across studies; with data from different countries contributing to all themes and sub-themes except for the sub-theme relating to infant feeding practices, a group which is understudied.

## Conclusions

This review has explored the ways in which food insecurity impacts children’s nutritional health and wellbeing from both caregivers’ and children’s own perspectives. We build on previous reviews, by focusing on using food as a lens to explore children’s lived experiences of disadvantage. It shows that children are aware of their family’s limited resources and are active in trying to help their families, and how food insecurity is an adverse physical, psychological, and social experience for children. It also highlights gaps in our knowledge about how food insecurity impacts children’s nutritional health and wellbeing. There is a need for more research with caregivers and the first 1001 days as there was an overall lack of studies specifically focusing on this life course stage which is important for lifelong health. This meant that the review was unable to fully explore the impact of food insecurity on infant feeding beliefs, styles, and practices. Further, few studies included children from minoritised ethnic communities, and gender differences in experiences are not explored in depth. Likewise, studies of the impact on children’s food and eating practices of living in temporary accommodation is under-researched. Policy makers should focus on including children as part of the decision-making process to mitigate food insecurity.

## Supporting information

S1 Checklist PRISMA 2020 checklist. (DOCX)

S1 File eMERGE reporting guidelines. (DOCX)

S2 File Search strategies adapted for each database. (DOCX)

S3 File Characteristics of included studies. (DOCX)

S4 File Critical appraisal skills programme (CASP) quality appraisal of included studies. (DOCX)
