# Supplementary material for: Children’s nutritional health and wellbeing in food insecure households in Europe: A qualitative meta-ethnography
Source: PLoS One. 2023 Sep 29;18(9):e0292178. doi: 10.1371/journal.pone.0292178 (PMC10540950; doi:10.1371/journal.pone.0292178)
Supplement: S1 File — (DOCX) [file pone.0292178.s002.docx]

**S2**: **eMERGE reporting guidelines**

| **No.** | **Criteria Headings** | **Reporting Criteria** | **Done** |
| --- | --- | --- | --- |
| **Phase 1 – Selecting meta-ethnography and getting started** | | |  |
| *Introduction* | | |  |
| 1 | Rationale and context for the meta-ethnography | Describe the gap in research or knowledge to be filled by the meta-ethnography, and the wider context of the meta-ethnography | 1-2 |
| 2 | Aim(s) of the meta-ethnography | Describe the meta-ethnography aim(s) | 2 |
| 3 | Focus of the meta-ethnography | Describe the meta-ethnography review question(s) (or objectives) | 2 & Protocol |
| 4 | Rationale for using meta-ethnography | Explain why meta-ethnography was considered the most appropriate qualitative synthesis methodology | 3 |
| **Phase 2 – Deciding what is relevant** | | | |
| *Methods* | | | |
| 5 | Search Strategy | Describe the rationale for the literature search strategy | 3-4 |
| 6 | Search Process | Describe how the literature searching was carried out and by whom | 3-4 |
| 7 | Selecting primary studies | Describe the process of study screening and selection, and who was involved | 4-5 |
| *Findings* | | | |
| 8 | Outcome of study selection | Describe the results of study searches and screening | 5-6 |
| **Phase 3 – Reading included studies** | | | |
| *Methods* | | | |
| 9 | Reading and data extraction approach | Describe the reading and data extraction method and process | 4-5 |
| *Findings* | | | |
| 10 | Presenting characteristics of included studies | Describe characteristics of the included studies | 5-6 |
| **Phase 4 – Determining how studies are related** | | | |
| *Methods* | | | |
| 11 | Process for determining how studies are related | Describe the methods and processes for determining how the included studies are related:  - Which aspects of studies were compared  AND  - How the studies were compared | 4 |
| *Findings* | | | |
| 12 | Outcomes of relating studies | Describe how studies relate to each other | 4 |
| **Phase 5 – Translating studies into one another** | | | |
| *Methods* | | | |
| 13 | Process of translating studies | Describe the methods of translation:  - Describe steps taken to preserve the context and meaning of the relationships between concepts within and across studies- Describe how the reciprocal and refutational translations were conducted- Describe how potential alternative interpretations or explanations were considered in the translations | 4 |
| *Findings* | | | |
| 14 | Outcome of translation | Describe the interpretive findings of the translation | 7-17 |
| **Phase 6 – Synthesising translations** | | | |
| *Methods* | | | |
| 15 | Synthesising process | Describe the methods used to develop overarching concepts (“synthesised translations”) Describe how potential alternative interpretations or explanations were considered in the synthesis | 4 |
| *Findings* | | | |
| 16 | Outcome of synthesis process | Describe the new theory, conceptual framework, model, configuration, or interpretation of data developed from the synthesis | 7-17 |
| **Phase 7 – Expressing the synthesis** | | | |
| *Discussion* | | | |
| 17 | Summary of findings | Summarise the main interpretive findings of the translation and synthesis and compare them to existing literature | 17-24 |
| 18 | Strengths, limitations and reflexivity | Reflect on and describe the strengths and limitations of the synthesis: - Methodological aspects—for example, describe how the synthesis findings were influenced by the nature of the included studies and how the meta-ethnography was conducted.- Reflexivity—for example, the impact of the research team on the synthesis findings | 24-25 |
| 19 | Recommendations and conclusions | Describe the implications of the synthesis | 25 |
