# Supplementary material for: Children’s nutritional health and wellbeing in food insecure households in Europe: A qualitative meta-ethnography
Source: PLoS One. 2023 Sep 29;18(9):e0292178. doi: 10.1371/journal.pone.0292178 (PMC10540950; doi:10.1371/journal.pone.0292178)
Supplement: S2 File — (DOCX) [file pone.0292178.s003.docx]

**S3: Search strategies adapted for each database**

**Web of Science**

(TI=(wom?n or female or mother* or matern* or mum or father* or dad or caregiver* or guardian* or parent* or preg* or postnatal or newborn or bab* or infan* or toddler or child* or preschool or adolescent or teen* or "young adult" or "lowest income group") ) OR (AB=(wom?n or female or mother* or matern* or mum or father* or dad or caregiver* or guardian* or parent* or preg* or postnatal or newborn or bab* or infan* or toddler or child* or preschool or adolescent or teen* or "young adult" or "lowest income group") )

AND ((TI=("food insecur*" or "food secur*" or "food poverty" or "food insufficienc*" or "food assistance" or "food depriv*" or poverty or "food bank*" or hunger or "access to food" or hardship or "food access") )OR (AB=("food insecur*" or "food secur*" or "food poverty" or "food insufficienc*" or "food assistance" or "food depriv*" or poverty or "food bank*" or hunger or "access to food" or hardship or "food access") )

AND (TS=(nutrition or "food practices" or "food preferences" or "healthy eating" or "family influences" or health or "feeding behaviour" or "feeding practices" or "food habit"s or diet or "diet quality" or "portion size" or breastfeeding or "complimentary feeding" or "maternal nutrition" or "child nutrition" or "infant food" or weight or obesity or "childhood obesity" or "toddler development" or growth or "growth trajectories") )

AND (TS=("qualitative research" or "grounded theory" or ethnograph* or phenomenolog* or feminis* or narrative or interview* or "focus group" or "case stud*" or anthrop* or observ* or "field notes" or biograph* or "life history" or photovoice or "photo elicitation" or autoethnograph* or "creative method" or "thematic analysis") )

*AND***LANGUAGE:** (English) *Indexes=SCI-EXPANDED, SSCI, A&HCI, CPCI-S, CPCI-SSH, ESCI Timespan=2008-2020*

**Scopus**

TITLE-ABS ( "food insecur*" OR "food secur*" OR "food poverty" OR "food insufficien*" OR "food assistance" OR "food depriv*" OR poverty OR "foodbanks" OR "food banks" OR hunger OR "food access*" )

AND TITLE-ABS("food insecur*" or "food secur*" or "food poverty" or "food insufficien*" or "food assistance" or "food depriv*" or poverty or "foodbanks" or "food banks" or hunger or "food access")

AND TITLE-ABS-KEY ( nutrition OR "food practices" OR "food preferences" OR "healthy eating" OR "feeding behaviour" OR "feeding practices" OR "food habits" OR diet OR "diet quality" OR "portion size" OR breastfeeding OR "complimentary feeding" OR weaning OR "maternal nutrition" OR "child nutrition" OR "infant food" OR weight OR obesity OR "childhood obesity" OR development OR growth )

AND TITLE-ABS-KEY("qualitative research" or "grounded theory" or ethnograph* or phenomenolog* or feminis* or narrative* or interview* or "focus group*" or "case stud*" or anthrop* or observ* or "field notes" or biograph* or "life history" or photovoice or "photo elicitation" or autoethnograph* or "creative method" or "thematic analysis")

( LIMIT-TO ( AFFILCOUNTRY,"United States" ) OR LIMIT-TO ( AFFILCOUNTRY,"United Kingdom" ) OR LIMIT-TO ( AFFILCOUNTRY,"Canada" ) OR LIMIT-TO ( AFFILCOUNTRY,"Australia" ) OR LIMIT-TO ( AFFILCOUNTRY,"Netherlands" ) OR LIMIT-TO ( AFFILCOUNTRY,"Germany" ) OR LIMIT-TO ( AFFILCOUNTRY,"France" ) OR LIMIT-TO ( AFFILCOUNTRY,"Sweden" ) OR LIMIT-TO ( AFFILCOUNTRY,"Italy" ) OR LIMIT-TO ( AFFILCOUNTRY,"Switzerland" ) OR LIMIT-TO ( AFFILCOUNTRY,"Spain" ) OR LIMIT-TO ( AFFILCOUNTRY,"Norway" ) OR LIMIT-TO ( AFFILCOUNTRY,"New Zealand" ) OR LIMIT-TO ( AFFILCOUNTRY,"Belgium" ) OR LIMIT-TO ( AFFILCOUNTRY,"Japan" ) OR LIMIT-TO ( AFFILCOUNTRY,"Denmark" ) OR LIMIT-TO ( AFFILCOUNTRY,"Finland" ) OR LIMIT-TO ( AFFILCOUNTRY,"Ireland" ) OR LIMIT-TO ( AFFILCOUNTRY,"Poland" ) OR LIMIT-TO ( AFFILCOUNTRY,"Israel" ) OR LIMIT-TO ( AFFILCOUNTRY,"Chile" ) OR LIMIT-TO ( AFFILCOUNTRY,"Austria" ) OR LIMIT-TO ( AFFILCOUNTRY,"Portugal" ) OR LIMIT-TO ( AFFILCOUNTRY,"Greece" ) OR LIMIT-TO ( AFFILCOUNTRY,"Czech Republic" ) OR LIMIT-TO ( AFFILCOUNTRY,"Hungary" ) OR LIMIT-TO ( AFFILCOUNTRY,"Slovakia" ) OR LIMIT-TO ( AFFILCOUNTRY,"Estonia" ) OR LIMIT-TO ( AFFILCOUNTRY,"Luxembourg" ) OR LIMIT-TO ( AFFILCOUNTRY,"Iceland" ) OR LIMIT-TO ( AFFILCOUNTRY,"Lithuania" ) OR LIMIT-TO ( AFFILCOUNTRY,"Slovenia" ) OR LIMIT-TO ( AFFILCOUNTRY,"Undefined" ) )

Limits 2008-current, English language

**CINHAL**

(MH "Women+") OR (MH "Expectant Mothers") OR (MH "Parents+") OR (MH "Fathers") OR "dad" OR (MH "Guardianship, Legal") OR (MH "Caregivers") OR (MH "Child+") OR (MH "Infant") OR (MH "Infant, Newborn+") OR (MH "Adolescence")

AND (MH "Food Security") OR (MH "Food Assistance") OR (MH "Hunger") OR (MH "Poverty") OR “"food pantry or food pantries or food bank or food banks or food access”

AND (MH "Nutrition") OR (MH "Diet") OR (MH "Food Intake") OR (MH "Infant Nutrition") OR (MH "Adolescent Nutrition") OR (MH "Child Nutrition") OR (MH "Food Preferences") OR (MH "Food Habits") OR (MH "Portion Size") OR (MH "Eating Behavior") OR (MH "Breast Feeding") OR (MH "Bottle Feeding") OR (MH "Infant Feeding") OR (MH "Body Weight") OR "growth and development"

AND (MH "Delphi Technique") OR (MH "Interviews") OR (MH "Narratives") OR (MH "Focus Groups") OR (MH "Observational Methods")) OR (MH "Qualitative Studies+")

Limits 2008-current, English language

**ASSIA**

(wom?n OR female OR mother* OR matern* OR mum OR father OR dad OR caregiver OR guardian OR parent* OR preg* OR postnatal OR newborn OR bab* OR infan* OR toddler OR child* OR preschool OR adolescent OR teen* OR "young adult" OR "lowest income group")

AND ("food insecur*" OR "food secur*" OR "food poverty" OR "food insufficien*" OR "food assistance" OR "food depriv*" OR "poverty" OR "foodbanks" OR "food banks" OR "hunger" OR "access to food" OR "food aid" or “food access*”)

AND (nutrition OR "food practices" OR "food preferences" OR "healthy eating" OR "feeding behaviour" OR "feeding practices" OR "food habits" OR diet OR "diet quality" OR "portion size" OR breastfeeding OR "complimentary feeding" OR weaning OR "maternal nutrition" OR "child nutrition" OR "infant food" OR weight OR obesity OR "childhood obesity" OR development OR growth)

AND ("qualitative research" OR "grounded theory" OR ethnograph* OR phenomenolog* OR feminis* OR narrative* OR interview* OR "focus group*" OR "case stud*" OR anthrop* OR observ* OR "field notes" OR biograph* OR "life history" OR photovoice OR "photo elicitation" OR autoethnograph* OR "creative method" OR "thematic analysis")

1 Jan 2008 to current, English Language

**Embase**

| 1 | poverty/ or food insecurity/ or food poverty.mp. or food availability/ |
| --- | --- |
| 2 | food assistance/ |
| 3 | food bank.mp. |
| 4 | food insufficiency.mp. |
| 5 | hunger/ or hunger.mp. |
| 6 | female/ |
| 7 | mother/ or parent/ or adolescent mother/ or expectant mother/ |
| 8 | adolescent father/ or father/ |
| 9 | parent/ or adolescent parent/ or adoptive parent/ or divorced parent/ or father/ or mother/ or separated parent/ or single parent/ |
| 10 | pregnancy/ |
| 11 | caregiver/ |
| 12 | legal guardian/ |
| 13 | newborn/ or infant/ |
| 14 | child/ or toddler/ or preschool child/ |
| 15 | young adult/ |
| 16 | adolescent/ |
| 17 | lowest income group/ |
| **18** | 6 or 7 or 8 or 9 or 10 or 11 or 12 or 13 or 14 or 15 or 16 or 17 |
| 19 | maternal nutrition/ or child nutrition/ or infant nutrition/ or adolescent nutrition/ or nutrition/ |
| 20 | diet/ or healthy diet/ |
| 21 | feeding behavior/ or eating habit/ or food preference/ or meal size/ or portion size/ |
| 22 | food practices.mp. |
| 23 | breast feeding/ or infant feeding/ |
| 24 | weaning/ |
| 25 | "physical constitution and health"/ or body constitution/ or body weight/ or health/ or wellbeing/ |
| 26 | adolescent obesity/ or childhood obesity/ or obesity/ or diet-induced obesity/ |
| 27 | child growth/ |
| 28 | child development/ |
| **29** | 19 or 20 or 21 or 22 or 23 or 24 or 25 or 26 or 27 or 28 |
| 30 | qualitative research/ |
| 31 | grounded theory/ |
| 32 | ethnography/ |
| 33 | phenomenology/ |
| 34 | feminism/ |
| 35 | narrative/ |
| 36 | interview/ or semi structured interview/ or structured interview/ or telephone interview/ or unstructured interview/ |
| 37 | focus group.mp. |
| 38 | case study/ |
| 39 | anthropology/ |
| 40 | thematic analysis/ |
| 41 | participant observation/ |
| 42 | field notes.mp. |
| 43 | participatory research/ |
| 44 | photo voice.mp. |
| 45 | photo elicitation.mp. |
| 46 | creative method.mp. |
| **47** | 30 or 31 or 32 or 33 or 34 or 35 or 36 or 37 or 38 or 39 or 40 or 41 or 42 or 43 or 44 or 45 or 46 |
| 48 | access to food.mp. |
| **49** | 1 or 2 or 3 or 4 or 5 or 48 |
| 50 | 18 and 29 and 47 and 49 |
| **51** | limit 50 to (human and english language and yr="2008 -Current") |

**OVID**

| 1 | Food Supply/ |
| --- | --- |
| 2 | food insecur*.mp. |
| 3 | Poverty/ or food poverty.mp. |
| 4 | Hunger/ or food insufficiency.mp. |
| 5 | Food Deprivation/ |
| 6 | Food Assistance/ |
| 7 | access to food.mp. |
| 8 | food bank*.mp. |
| 9 | 1 or 2 or 3 or 4 or 5 or 6 or 7 or 8 |
| 10 | Pregnancy/ or Female/ or wom?n.mp. |
| 11 | exp parents/ or single-parent family/ |
| 12 | Legal Guardians/ |
| 13 | Caregivers/ |
| 14 | adult children/ or age groups/ or adolescent/ or adult/ or child/ or infant/ or infant, newborn/ |
| 15 | lowest income group.mp. |
| 16 | 10 or 11 or 12 or 13 or 14 or 15 |
| 17 | feeding behavior/ or bottle feeding/ or breast feeding/ or food preferences/ |
| 18 | infant feeding.mp. |
| 19 | "diet, food, and nutrition"/ or food/ or diet/ or diet, healthy/ or portion size/ or serving size/ |
| 20 | child nutrition.mp. |
| 21 | exp Body Weight/ |
| 22 | malnutrition/ or overnutrition/ or obesity/ or obesity, abdominal/ or obesity, maternal/ or obesity, morbid/ or pediatric obesity/ |
| 23 | "growth and development"/ or growth/ or human development/ |
| 24 | 17 or 18 or 19 or 20 or 21 or 22 or 23 |
| 25 | grounded theory/ or qualitative research/ |
| 26 | ethnograph*.mp. |
| 27 | phenomenology.mp. |
| 28 | feminis*.mp. |
| 29 | narrative.mp. |
| 30 | interview/ |
| 31 | Focus Groups/ |
| 32 | case stud*.mp. |
| 33 | Anthropology, Cultural/ |
| 34 | observ*.mp. |
| 35 | field notes.mp. |
| 36 | biograph*.mp. |
| 37 | life history.mp. |
| 38 | Community-Based Participatory Research/ or photovoice.mp. |
| 39 | photo elicitation.mp. |
| 40 | autoethnography.mp. |
| 41 | creative method.mp. |
| 42 | thematic analysis.mp. |
| 43 | 25 or 26 or 27 or 28 or 29 or 30 or 31 or 32 or 33 or 34 or 35 or 36 or 37 or 38 or 39 or 40 or 41 or 42 |
| 44 | 9 and 16 and 24 and 43 |
| 45 | limit 44 to (English language and humans and yr="2008 -Current") |
