# Supplementary material for: Children’s nutritional health and wellbeing in food insecure households in Europe: A qualitative meta-ethnography
Source: PLoS One. 2023 Sep 29;18(9):e0292178. doi: 10.1371/journal.pone.0292178 (PMC10540950; doi:10.1371/journal.pone.0292178)
Supplement: S3 File — (DOCX) [file pone.0292178.s004.docx]

**S4: Characteristics of included studies**

**Table 1: Caregiver’s perspectives only**

| Study | Country of study | Focus^ | Study aim | Method | Setting | Participants | SES | Measure of food insecurity |
| --- | --- | --- | --- | --- | --- | --- | --- | --- |
| Hayter et al., (2015) | England | Secondary | To explore parents’ perceptions of feeding their pre-school children in two low-income populations in England | 4 focus groups  Family interviews | Community Children’s Centres  Islington, inner city urban London  Cornwall, rural county in Southwest England | - N = 33 focus group participants & 6 family interview participants (36 mothers and 3 fathers) - Age = Children between 18 months – 5 years - Ethnicity = Not stated | No socio-economic data was collected, but all children’s centres were in deprived areas, which target deprived families, families considered vulnerable by staff were invited to participate | Not reported |
| Lovelace and Rabiee-Khan (2015) | England | Secondary | To explore the influences on the diets of young children in families on low income | Semi-structured interviews | West Midlands, UK | - N = 12 (10 mothers + 2 fathers present at interview) - Age = Children 2 – 37 months (average age 22 months) - Ethnicity = Not stated | Low income (did not own their own home, were in receipt of income support and/or qualifying for HSV* | In receipt of or qualifying for HSV |
| Nielsen et al., (2015) | Denmark | Secondary | To aims provide insight into typical patterns of coping among budget-restricted households | Interviews | Homes of participants  Denmark | - N = 30 Danish individuals (2 low-income women) - Age = Not stated - Ethnicity = Not stated | Women with low-income  Women had implemented changes to food practices due to economic restraint | Not reported |
| Spencer (2015) | Scotland | Primary | How does urban inequality and deprivation affect the way those facing these issues source food and how does this affect their food choices? How do other modern issues relating to deprivation affect diet and their meanings and interpretations of food e.g. being in receipt of benefits or having to use food banks as a source of food? | Semi-structured interview | Community setting  Aberdeen | - N = 15 (9 female (2 low-income mothers) - Age = 25-45 years - Ethnicity = Not stated | 2 mothers single, unemployed living in 10% most deprived area of Scotland, renting housing from council, 1 woman part-time employment, divorced, renting from housing association | Not reported |
| Condon and McClean, (2016) | England | Secondary | To explore the barriers and facilitators to maintaining pre-school children' health amongst migrant families in the UK | 5 focus groups with parents (in first language of participants) | Community  England | - N = 28 (22 mothers, 6 fathers) - Age = All children under 6 years - Ethnicity =.7 Romanian, 6 Roma, 6 Polish, 5 Somali, 4 Pakistani parents | Families recruited from inner-city areas in the most deprived 10% in England (2 families living in temporary accommodation) | Not reported |
| Canton, (2018) | England | Secondary | To explore lone mothers’ experiences of economic crisis and austerity, examining the ways in which their social relationships help them cope and adapt | Semi-structured interviews | Bath or Bristol Southwest of England | - N = 30 lone mothers - Age = 21-52 years - Ethnicity = Not stated | 15 mothers were in paid employment  Just over half of the participants had an undergraduate degree or more | Not stated |
| Jolly (2018) | England | Secondary | (1) To understand the experiences of food poverty for families who were at risk of destitution because of their immigration status. (2) To identify transferable learning for practitioners to improve social work and social care practice with this service user group. | Series of semi-structured interviews | Play sessions at the project where the researcher worked  Birmingham, England, UK | - N = 7 parents from 6 families - Age = Not stated - Ethnicity = Variety of backgrounds and immigration status. 1 Eastern European accession country, 1 asylum seeker, 1 with discretionary leave to stay in the UK, 4 undocumented after overstaying their visas | All had experienced destitution and were in receipt of services by local authority for their children  Living in a range of housing; privately rented, temporary bed and breakfast, sofa-surfing | Not reported |
| Zamora-Sarabia et al., (2019) | Spain | Primary | To understand the factors which are perceived by parents attending the foodbank to shape a) the health of their children and b) the possibility for childcare in a context of poverty and food insecurity | 7-month participant observations, two researchers, two days a week  In-depth interviews | Foodbank  District of Tetuán, Madrid | - N = 15 (10 mothers, 5 fathers) Among them a total of 22 children (14 girls, 9 boys) - Age = Children aged 1.5-17 years - Ethnicity = Not stated | 7 mothers, 5 fathers unemployed, 3 mothers employed (for a max. of 438 euros per month).  3 mothers, 1 father from Spain, 7 mothers, 4 fathers immigrants | Accessing a foodbank |
| Share (2019) | Ireland | Primary | To understand the dynamic relationship between people, space and food in the particular context of homeless accommodation provision | Interviewer-administered background survey  In-depth photo elicitation interview | Dublin, Ireland | - N = 10 parents / families (4 male, 6 female) - Age = Mean age 34.4 years - Ethnicity = Not stated | 4 parents in couple households, 6 in single-parent households  4 parents in hostel for homeless, 3 parents in budget B&B for homeless & tourists, 2 parents in commercial hotel geared for tourists, 1 parent in budget hotel for homeless | Not reported |
| Power et al., (2021)* | England | Primary | To explore lived experiences of food insecurity and underlying drivers of diet quality among low-income families, drawing upon two years of participatory research with families of primary school age children, 4-11 years | Focus groups | Community centre or café  York, North of England | - N = 22 (19 were female) - Age = Not stated - Ethnicity = Not stated | Participants self-identified as parents or caregivers living on a low income | Not reported |

^ Primary = studies with food insecurity as the focus. Secondary = studies where food insecurity was discussed as part of the wider research

* FSM = Free School Meals, USDA HFSSM = United States Department of Agriculture Household Food Security Survey Module, HSV = Healthy Start Vouchers, IMD = Indices of Multiple Deprivation

**Table 2:** **Characteristics of included studies – both caregiver’s and children’s perspectives**

| Study | Country of study | Focus^ | Study aim | Method | Setting | Participants | SES | Measure of food insecurity |
| --- | --- | --- | --- | --- | --- | --- | --- | --- |
| Hall et al., (2013) | England | Primary | 1) To understand the lived experiences of children and their families against a backdrop of rising food prices; and,  2) The positive steps that families have taken to meet the challenge of food affordability and support themselves, in addition to the experiences of families who are not able to cope. | Family case studies  Participatory photography | In-home case study visits  London, UK | - N = 5 family case studies (3 single mothers, 4 parents) - Age = Not stated - Ethnicity = Not Stated | 4 families in receipt in FSM, 1 family eligible but child refuses FSM  Low-income families (Single parents; 1 employed full-time, 1 part-time, 1 unemployed, coupled parents each with 1 unemployed and 1 employed part-time or full-time) | Family structure + household income + eligibility for FSM |
| Hall and Perry (2013) | England | Secondary | To understand and convey: (1) the lived experience of families against a backdrop of austerity; the various impacts of austerity on family life (2) what matters to families and supports them under conditions of austerity, with a particular focus on family finances and wellbeing | Semi-structured in-depth interviews  Self-completion diaries  Participatory photographs | Not Stated  England | - N = 11 families (7 coupled parents, 4 single parent) - Age = Not Stated - Ethnicity = Not Stated | 4 families lived in urban areas, 3 sub-urban, 4 rural areas  5 families with mortgage, | Not reported |
| Garthwaite et al., (2015) | England | Primary | To examine the relationship between ill health and food insecurity among foodbank users in the UK | Participant observations  Interviews | Foodbank  Stockton-on-Tees, Northeast England, UK | - N = 42 foodbank users (20 female, 22 male) - Age = 18-51 years (Child = 11 years) - Ethnicity = Not stated | All participants were on low income or accessing a form of social security | Accessing a food bank |
| Dalma et al., (2016) | Greece | Secondary | To explore the perceptions of parents and students towards healthy eating and related barriers, and their experience of a school feeding programme | 20 focus groups carried out separately with children and parents | Schools  Province of Attica (Athens is its capital) | - N = 44 parents, 98 children - Age = Children 8-12 years & junior high students (age not specified) - Ethnicity = Not stated | Details for children not given but students were attending elementary and secondary public schools in low socio-economic status regions of Greece | Incidence of food insecurity and other indicators of poverty provided by principal upon school selection – details not stated |
| Purdam et al., (2016) | England | Primary | To understand the concerns food bank users have when visiting a food bank. To explore the sustainability of local voluntary-led food aid policy models | Four case studies  Interviews | Foodbanks in Northwest England, UK | - N = 35 (23 women, 7 men, 2 were couples, 1 child) - Age = mean age 51 years, child 10 years - Ethnicity = Not stated | 25 participants had children  3 employed, 31 in receipt of different welfare benefits or waiting for application approval, some paying benefit sanctions | Accessing a foodbank |
| O’Connell and Brannen (2021) | England, Portugal, Norway | Primary | To examine how experiences of food poverty are shaped by social contexts and social positionings | In-depth interviews  Tour of kitchen with follow-up interview with parents  Vignettes  Photo-elicitation interviews | Participant’s homes  Inner London or coastal town in South East of England  Urban / Sub-urban areas of Lisbon or Rural areas further away  Urban areas across Oslo or Rural / semi- rural areas in non- urban Eastern Norway | - N= 145 children and young people, 133 parents or caregivers (mostly mothers) - Age = children and young people 11-16 years, parents or caregivers age not stated - UK = 45 families and 51 children - Portugal = 45 families and 46 children - Norway = 43 families and 48 children | Families were all deemed low-income by themselves i.e. their income was below what they needed  41 UK families, 44 Portuguese families and 6 Norwegian families met the relative low-income measure employed as poverty (income decile 1 or 2) | Case studies reported were all experiencing or had recently experienced a food shortage |

^ Primary = studies with food insecurity as the focus. Secondary = studies where food insecurity was discussed as part of the wider research

* FSM = Free School Meals, USDA HFSSM = United States Department of Agriculture Household Food Security Survey Module, HSV = Healthy Start Vouchers, IMD = Indices of Multiple Deprivation

**Table 3: Characteristics of included studies – children’s perspectives only**

| Study | Country of study | Focus^ | Study aim | Method | Setting | Participants | SES | Measure of food insecurity |
| --- | --- | --- | --- | --- | --- | --- | --- | --- |
| Fairbrother et al., (2012) | England | Primary | To explore children’s understanding of family finances and how they perceive this to relate to eating healthily | Photo-elicited interviews  Debate within groups  Follow- up interviews | Photo-elicited interviews and debates within schools  Follow-up interviews at child’s home  North of England | - N = 53 (8 follow-up interviews at home) (24 children in socioeconomic disadvantaged school and 29 children in advantaged school) - Age = 9-10 years - Ethnicity = all disadvantaged children were White British | Details for children not given but children attending two socioeconomically contrasting schools in urban neighbourhoods, determined by eligibility for FSM* and local area knowledge | Eligibility for FSM* - details not stated |
| Harvey et al., (2016) | England | Primary | To gain an understanding of London families' experiences of food insecurity by describing its impact from parents' perspectives and obtaining children's narratives ^^ | Semi-structured interviews | Community, Arches II Centre (part of Kids Company Charity)  South Lambeth, London | - N = 19 children (from 14 families) (mix of male & female, small majority female, 58%) - Age = 5-11 years (median 9 years) - Ethnicity = Not stated | Children from deprived households as accessing Kids Company Charity services | 16 children very low food security, 3 children low food security  Parents completed a questionnaire with USDA HFSSM |
| Laverty (2019) | England | Primary | To explore how children and young people experience food insecurity, particularly outside of the home and school, and the informal practices they use to manage food insecurity | 14-month participant observations  2 focus groups | Community Youth Centre  North of England | - N = 30 young people observed - 20 participants focus groups (12 boys, 8 girls) - Age = 11-25 years - Ethnicity = Not stated | Details for children not given but youth centre situated in North of England in one of the most deprived neighbourhoods by IMD* | ~60% of attendees at youth centre eligible for FSM* |

^ Primary = studies with food insecurity as the focus. Secondary = studies where food insecurity was discussed as part of the wider research

* FSM = Free School Meals, USDA HFSSM = United States Department of Agriculture Household Food Security Survey Module, HSV = Healthy Start Vouchers, IMD = Indices of Multiple Deprivation
