# Supplementary material for: Children’s nutritional health and wellbeing in food insecure households in Europe: A qualitative meta-ethnography
Source: PLoS One. 2023 Sep 29;18(9):e0292178. doi: 10.1371/journal.pone.0292178 (PMC10540950; doi:10.1371/journal.pone.0292178)
Supplement: S4 File — (DOCX) [file pone.0292178.s005.docx]

**S5: Critical Appraisal Skills Programme (CASP) quality appraisal of included studies.**

| CASP Question | Canton, 2018 | Condon and McClean, 2017 | Dalma et al., 2016 | Fairbrother et al., 2012 | Garthwaite et al., 2015 | Hall et al., 2013 | Hall & Perry, 2013 | Harvey et al., 2016 |  | Hayter et al., 2015 | Jolly, 2018 |  |
| --- | --- | --- | --- | --- | --- | --- | --- | --- | --- | --- | --- | --- |
| Clear relevant aim? | Yes | Yes | Yes | Yes | Yes | Yes | Yes | Yes |  | Yes | Yes |  |
| Appropriate methodology? | Yes | Yes | Yes | Yes | Yes | Yes | Yes | Yes |  | Yes | Yes |  |
| Appropriate research design? | Yes | Yes | Yes | Yes | Yes | Yes | Yes | Yes |  | Yes | Yes |  |
| Appropriate recruitment strategy? | Yes | Yes | Yes | Yes | Can’t tell | Can’t tell | Yes | Yes |  | Yes | Yes |  |
| Appropriate data collection method? | Yes | Yes | Yes | Yes | Yes | Yes | Yes | Yes |  | Yes | Yes |  |
| Reflexivity discussed? | Can’t tell | Yes | Can’t tell | Yes | Yes | No | No | Yes |  | Can’t tell | Can’t tell |  |
| Ethical issues been considered? | Yes | Yes | Yes | Yes | Yes | No | Can’t tell | Yes |  | Can’t tell | Can’t tell |  |
| Sufficiently rigorous data analysis methods? | Can’t tell | Yes | Yes | Yes | Yes | No | No | Yes |  | Yes | Can’t tell |  |
| Clear statement of finding? | Yes | Yes | Yes | Yes | Yes | Yes | Yes | Yes |  | Yes | Yes |  |
| Valuable research question? | Yes | Yes | Yes | Yes | Yes | Yes | Yes | Yes |  | Yes | Yes |  |
| Score  Quality | **18**  **Good** | **20**  **High** | **19**  **Good** | **20**  **High** | **19**  **Good** | **13**  **Low** | **15**  **Low** | **20**  **High** |  | **18**  **Good** | **17**  **Good** |  |

**S5: Critical Appraisal Skills Programme (CASP) quality appraisal of included studies (continued)**

| CASP Question | Laverty, 2019 | Lovelace and Rabiee-Khan, 2015 | Nielsen et al., 2015 | O’Connell and Brannen, 2021 | Power et al., 2021 | Purdam et al.,2016 | Share, 2019 | Spencer, 2015 | Zamora-Sarabia et al., 2019 |
| --- | --- | --- | --- | --- | --- | --- | --- | --- | --- |
| Clear relevant aim? | Yes | Yes | Yes | Yes | Yes | Yes | Yes | Yes | Yes |
| Appropriate methodology? | Yes | Yes | Yes | Yes | Yes | Yes | Yes | Yes | Yes |
| Appropriate research design? | Yes | Yes | Yes | Yes | Yes | Yes | Yes | Yes | Yes |
| Appropriate recruitment strategy? | Yes | Yes | Yes | Yes | Yes | Can’t tell | Yes | Yes | Yes |
| Appropriate data collection method? | Yes | Can’t tell | Yes | Yes | Yes | Yes | Yes | Yes | Yes |
| Reflexivity discussed? | Yes | Yes | No | Yes | Yes | Can’t tell | Yes | Can’t’ tell | Yes |
| Ethical issues been considered? | Yes | Yes | Yes | Yes | Yes | Can’t tell | Yes | Can’t tell | Yes |
| Sufficiently rigorous data analysis methods? | Yes | Yes | Yes | Yes | Yes | No | Yes | Yes | Yes |
| Clear statement of finding? | Yes | Yes | Yes | Yes | Yes | Yes | Yes | Yes | Yes |
| Valuable research question? | Yes | Yes | Yes | Yes | Yes | Yes | Yes | Yes | Yes |
| Score  Quality | **20**  **High** | **19**  **Good** | **18**  **Good** | **20**  **High** | **20**  **High** | **15**  **Low** | **20**  **High** | **18**  **Good** | **20**  **High** |
